# *Trichoderma harzianum*-Mediated ZnO Nanoparticles: A Green Tool for Controlling Soil-Borne Pathogens in Cotton

**DOI:** 10.3390/jof7110952

**Published:** 2021-11-10

**Authors:** Shimaa A. Zaki, Salama A. Ouf, Fawziah M. Albarakaty, Marian M. Habeb, Aly A. Aly, Kamel A. Abd-Elsalam

**Affiliations:** 1 Botany and Microbiology Department, Faculty of Science, Cairo University, Giza 12613, Egypt; shim.shimshim@yahoo.com (S.A.Z.); salama@sci.cu.edu.eg (S.A.O.); 2 Plant Pathology Research Institute, Agricultural Research Centre, Giza 12619, Egypt; marianmonir12@gmail.com (M.M.H.); aly.a.a@post.com (A.A.A.); 3 Department of Biology, Faculty of Applied Science, Umm Al-Qura University, Makkah Al Mukarramah P.O. Box 715, Saudi Arabia

**Keywords:** zinc oxide nanoparticles, *Gossypium barbadense*, *Fusarium* sp., *Rhizoctonia solani*, *Macrophomina phaseolina*

## Abstract

ZnO-based nanomaterials have high antifungal effects, such as inhibition of growth and reproduction of some pathogenic fungi, such as *Fusarium* sp., *Rhizoctonia solani* and *Macrophomina phaseolina*. Therefore, we report the extracellular synthesis of ZnONPs using a potential fungal antagonist (*Trichoderma harzianum*). ZnONPs were then characterized for their size, shape, charge and composition by visual analysis, UV–visible spectrometry, X-ray diffraction (XRD), Zeta potential, transmission electron microscopy (TEM), scanning electron microscopy (SEM) and energy-dispersive X-ray analysis (EDX). The TEM test confirmed that the size of the produced ZnONPs was 8–23 nm. The green synthesized ZnONPs were characterized by Fourier transform infrared spectroscopy (FTIR) studies to reveal the functional group attributed to the formation of ZnONPs. For the first time, trichogenic ZnONPs were shown to have fungicidal action against three soil–cotton pathogenic fungi in the laboratory and greenhouse. An antifungal examination was used to evaluate the bioactivity of the mycogenic ZnONPs in addition to two chemical fungicides (Moncut and Maxim XL) against three soil-borne pathogens, including *Fusarium* sp., * Rhizoctonia solani* and *Macrophomina phaseolina*. The findings of this study show a novel fungicidal activity in in vitro assay for complete inhibition of fungal growth of tested plant pathogenic fungi, as well as a considerable reduction in cotton seedling disease symptoms under greenhouse conditions. The formulation of a trichogenic ZnONPs form was found to increase its antifungal effect significantly. Finally, the utilization of biocontrol agents, such as *T. harzianum*, could be a safe strategy for the synthesis of a medium-scale of ZnONPs and employ it for fungal disease control in cotton.

## 1. Introduction

Cotton (*Gossypium barbadense* L.) is a globally important crop that is extensively produced and traded, as well as one of Egypt’s most valuable export crops [1]. Diseases of cotton seedlings are a worldwide problem caused by pathogenic soil-borne fungi. *Fusarium* spp. and *Rhizoctonia solani* are among the most pathogenic fungi present in cotton-producing regions in Egypt [2]. *R. solani* Kuhn, an anamorph of *Thanatephorus cucumeris* (Frank.) Donk [3], can cause pre-or post-emergence damping-off, seedling blight and root rot in cotton seedlings. *Fusarium* spp. are frequently obtained from infected cotton roots and classified as cotton seedling root pathogens [4]. *M. phaseolina* (*Tassi*) Goid infects over 100 families and 500 plant species all over the world [5,6]. *M. phaseolina* can cause charcoal rot in an abroad range of crops, such as sorghum, soybean, cotton, bean and corn, when conditions are favorable [7].

Bio-based NP synthesis has received a lot of interest in the last five years. It has eliminated difficult procedures necessary for NPs production utilizing microorganisms, such as fungi, bacteria and yeast, such as microbial cell culture upkeep, prolonged incubation time, several purification steps and so on [8]. Mycogenic nanoparticles offer advantages, including the formation of a capping from fungal biomolecules, which provides stability and can contribute to various biological activities [9]. ZnONPs were synthesized from fungal secondary metabolites of three monocultures of *Trichoderma* species including, *T. harzianum* and *T. reesei*. ZnONPs were biogenically produced using a cell filtrate of a strain of *T. harzianum* as a reducer and stabilizer agent [10]. Nevertheless, the microbial synthesis of ZnNPs remains unexplored [11]. *Monoascus purpureus*-mediated zinc oxide nanoparticles showed potent antifungal activity against six species of the most common food spoilage fungi [12].

Sustainable nanomaterials have become a promising option to control plant pathogenic fungi that are responsible for diseases in different crops. Crops treated with safe nano-fungicides will acquire additional value because they are free of chemicals and effective at low doses [9]. They reduce food and feed spoilage and fungal pathogens and help protect human health and sustain the universal demand for high product quality [13,14]. Because of the broad range of uses of zinc oxide nanoparticles (ZnONPs), such as smart UV sensors, they have piqued researchers’ attention [15], targeted drug delivery [16], antioxidant activity [17] biosensors [18], environmental remediation [19] and as a drought-tolerant agent as well as nutrient supply of crops [20]. Moreover, ZnONPs are characterized to be efficient against pathogenic fungi, mostly by their antimicrobial properties according to their photo-oxidizing and photocatalytic effects and considering infection control for the plant host [21]. Recently detailed reviews introduced the preparation methods and antifungal properties of ZnONPs and their possible antifungal mechanisms for plant diseases management and to improve food quality [22,23]. Under in vitro conditions, the biosynthesis of ZnONPs produced from *Trichodermas* spp. was used to suppress the development of *Xanthomonas oryzae* pv. *oryzae* [10]. An interactive protective impact of ZnONPs on seedling spray/seed soak followed by seedling and biocontrol treatments, *T. harzianum*, enhanced plant resistance to *R. solani*, the causal organisms of sunflower seedlings damping-off [24].

Antibacterial, antifungal, antiviral and anti-toxigenic activities against a range of phytopathogens may be achieved using zinc-based nanomaterials, which have targeted antimicrobial capabilities and low to negligible phytotoxic activities (Khan et al., 2021) [25]. Several applications methods may be used to employ these formulations in open fields or under greenhouse environments [22,23,24]. The use of fungi in the biogenic synthesis of ZnONPs has several benefits, including the production of a capping from fungal biomolecules, which provides stability and can contribute to different biological activities, such as the development of safe nanofungicides. Limited reports have used a *T. harzianum* cell filtrate for the production of ZnONPs as only a reducer and stabilizer agent [26]. Therefore, the main aims of the present study were to 1)synthesis a novel trichogenic-ZnONPs using an easy, eco-friendly, environmentally safe and costless approach, employing fungal metabolites from *T. harzianum* strains as a reducing agent and stabilizer to synthesize ZnONPs;2) characterize the synthesized ZnONPs to confirm synthesis, structure, size and NPs morphology; 3) investigate the in vitro and in vivo antifungal activity of mycogenic ZnONPs against soil-borne pathogenic fungi including *R. solani*, *Fusarium* sp. and *M. phaseolina* isolated from infected soil in cotton-growing areas.

## 2. Materials and Methods

### 2.1. Preparation of Trichoderma Isolates Culture

*Trichoderma* sp. isolates were isolated from healthy cotton root rhizosphere soils. Various soil dilutions were cultivated on Rose Bengal Medium (Sigma–Aldrich, St. Louis, MO, USA) [27]. After 48 h, *Trichoderma* colonies were collected and cultivated on potato dextrose agar (PDA) (Sigma–Aldrich, St., Louis, MO, USA) media. Single spore isolation was used to isolate putative *Trichoderma* colonies [28]. To obtain a pure isolate, a single spore was transferred to a PDA medium. *Trichoderma* species were identified based on morphology technique in Assiut University Mycology Center (AUMC), Assuit, Egypt.

### 2.2. Preparation of Cultural Extract

The fungi were allowed to grow aerobically in a liquid medium containing KH_2_PO_4_ (7 g/L); K_2_HPO_4_ (2 g/L); MgSO_4_·7H_2_O (0.1 g/L); (NH_2_)SO_4_ (1 g/L);yeast extract (0.6 g/L); glucose (10 g/L), to prepare the biomass for biosynthesis experiments. The growing cultures were incubated in an orbital shaker and agitated at 150 rpm at 27 °C. The produced biomass was collected by sieving through a plastic sieve after 72 h of growth. After that, the biomass was thoroughly washed with sterile distilled water to remove any remaining broth medium components. At 28 °C for 48 h, *Trichoderma* biomass (20 g) was transferred to an Erlenmeyer flask containing 100 mL sterile deionized water. The biomass was agitated after incubation and the filtrate was obtained by filtering it using Whatman (Sigma–Aldrich, St., Louis, MO, USA) filter paper #1 [29].

### 2.3. Synthesis of Zinc Oxide Nanoparticles

Fifty millilitres Zn(CH_3_COO)_2_.2H_2_O salt (Molecular Biology Grade, Merck, Kenilworth, NJ, USA) was applied to 50mL fungal filtrate and incubated for 72 h at 150 rpm in an orbital shaker. A fungal biomass filtrate without the zinc acetate dihydrate solution served as a positive control, while the zinc acetate dihydrate with cell-free filtrate served as a negative control. After centrifugation at 10,000 rpm for 10 min, the pellet aggregated at the bottom of the flask was removed from the filtrate and lyophilized [30]. ZnONPs were dried overnight at 60 °C in an oven and used for further research to evaluate fungicidal activity. For NPs characterization, the developed ZnONPs were subjected to a variety of instrumental analytical techniques to describe physico-chemical proporties.

### 2.4. Characterization of Nanoparticles

#### 2.4.1. Ultraviolet-Visible Spectrophotometer Analysis

A UV-vis spectrophotometer (T80 UV/vis spectrophotometer, PG Instruments Limited, Lutterworth, UK) was used to test the formation of reduced nanoparticles in colloidal solution. The supernatants’ absorption spectra were measured between 200 and 800 nm. The formation of ZnONPs was detected by periodic sampling of aliquots (1 mL) of the aqueous portion in the range of 0 to 1100 nm using an ultraviolet-visible spectrophotometer after 2, 4 and 7 days. Distilled water was used as a blank.

#### 2.4.2. Transmission Electron Microscopy (TEM)

Powder samples were put into a mortar before being mounted on a 200-mesh copper specimen grid with a film coating. At an accelerating voltage of 80 kV, TEM micrographs were taken on a Carl Zeiss Leo 912 AB OMEGA electron microscope (Carl Zeiss AG, Jena, Germany). After a drop of liquid ZnONPs was dried on the carbon-coated copper grids, a sample for analysis was prepared. Until loading onto a specimen holder, desiccators were used to dry TEM grid samples and keep them under vacuum. Image J 1.45 s software was used to assess the particle size distribution of nanoparticles.

#### 2.4.3. Zeta Potential

A sample was prepared by dissolving ZnONPs powder in deionized water, then the sample was sonicated for 10 min using the Q500 sonicator. The zeta potential of an aqueous solution of ZnONPs was measured in Folded Capillary cell (DTS1070) with pH ranging from 2 to 11 by applying ±65 V across the electrodes by Zetatrac equipped with Mic rotrac FLEX Operating Software on Mansettingnano (Malvern Instruments, South borough, MA, USA).

#### 2.4.4. X-ray Diffraction (XRD)

X-ray Diffraction (XRD) analysis was used to examine the structure of powder nanoparticles. Cu Kα radiation (*λ* = 1.54 Å) was used in the scattering range(2θ) of 0 to 80° at a scan rate of 0.03S1 on a D8-A25-Advance diffractometer (Bruker, Karlsruhe, Germany). As an internal standard for calibration, a standard silicon sample was used.

#### 2.4.5. Scanning Electron Microscope (SEM)

Scanning electron microscopical analysis was made using a Tescan SEM (Tescanvega 3 SBU, Czech Republic) at an accelerating voltage of 20 kV. Samples were mounted on aluminium microscopy stubs using carbon tape, then coated with gold (Au) for 120 s using a Quorum Techniques Ltd. sputter coater (Q150t, Lewes, UK).

#### 2.4.6. Energy Dispersive X-ray (EDX) Spectroscopy

On a JEOL(JEM-1230) electron microscope (Jeol, Tokyo, Japan), a drop of ZnONPs was put on carbon-coated copper grids and allowed to sit for 2 min and the excess solution was extracted with blotting paper and allowed to dry at room temperature.

#### 2.4.7. Fourier TransformInfrared Spectroscopy (FTIR)

FTIR spectra were performed on a JASCO-4700 FTIR Spectrometer (Laser Spectroscopy Labs, UCI, Irvine, CA, USA) to detect the possible functional groups in biomolecules present in the fungal extract.

### 2.5. In Vitro Antifungal Activity of Synthesized Nanoparticles

To evaluate the antifungal effect of nanoparticles in vitro, *R. solani* (Rs9), *Fusarium* sp. (F10) and *M. phaseolina* isolate (M4) were grown in PDA medium at 35 °C for 7 days. Then, freshly prepared PDA containing different concentrations of synthesized ZnONPs (20, 40 and 100 μg/mL) were added. ZnONPs solutions were put in an ultrasonic bath for 15 min at a sonicating frequency of 37 kHz (Elmasonic S60, Elma, Singen, Germany) to disrupt nanoparticle aggregations. After the fungal media cooled to about 45 °C, the sonicated NPs were inoculated into the media, then the fungal culture medium was poured into Petri dishes. Five-millimetre disks of fungal inoculum were cut with a cork borer and inoculated at the center of the 9-cm-diameter Petri dish, incubated at 35 °C for 5–7 days. PDA plates free from ZnONPs cultured under the same conditions were used as controls. The linear growth of the fungi was measured [31].

### 2.6. Antifungal Activity under Greenhouse Conditions

In a greenhouse experiment, the effects of synthesized ZnONPs were evaluated against *R. solani* (RS9), *Fusarium* sp. (F10) and *M. phaseolina* (M4) on cotton cultivars Giza90 and Giza94. The pots, containing the autoclaved soil, were infested with two-week-old pathogen-sorghum cultures of *R. solani* (RS9), *Fusarium* sp. (F10) and *M. phaseolina* (M4) at a rate of 1, 5 and 50 g/kg soil, respectively. Cotton cultivars Giza90 and Giza94 seeds were surfaces sterilized with 10% sodium hypochlorite for 2 min before being washed in four changes of sterilized water. The sterilized seeds were subsequently immersed in a suspension of ZnONPs at 100 and 200 μg/mL concentrations for 12 h under static circumstances. The tested fungicides (Moncut (2 g/kg seeds) and Maxim XL (2 mL/L)) were added to slightly moist seeds of cotton cultivars Giza90 and Giza94. Table 1. Infested soil was poured into 15-cm pots, with 10 seeds sown in each pot. Sterilized sorghum grains were mixed fully with soil cotton seeds only in the control treatments. In infested control, infested soil at the rate of 1 g/kg of soil *R. solani* (RS9) and 50 g/kg of soil for *Fusarium* sp. (F10) and *M. phaseolina* (M4) with cotton seeds without any treatment was applied. At 28 °C, pots were dispersed on greenhouse benches randomly in a complete block design. There were three replicates (pots) for each treatment. Forty-five days after planting, plant height (cm/plant), dry weight (g/plant) and survival percentages were all measured [32].

### 2.7. Statistical Analysis

Data were subjected to statistical analysis of variance (ANOVA) via MSTAT-C software. The mean differences were compared by the least significant difference (LSD) test at *p* ≤ 0.05.

## 3. Results

### 3.1. Trichoderma Isolates

A total of 50 *Trichoderma* strains (TC1–T50) were obtained from 22 soil samples. On the PDA medium, the colony’s growth speed, conidiospore color, wheel pattern and pigment secretion were all studied. Then, they were identified by morphological methods, which identified 6 *Trichoderma* species.

### 3.2. Trichogenic Nanoparticles Synthesis

The synthesis of ZnONPs was detected by UV-vis and from all the strains screened, only four had the aptitude to synthesize ZnONPs. Three *Trichoderma* species (Tvivi, TC34 and TC28) were used for the biosynthesis of stable ZnONPs. Filtrates from each fungal strain were incubated with zinc acetate dihydrate for 72 h under dark conditions at 28 °C with agitation.

### 3.3. Physiochemical Characterization

#### 3.3.1. UV-Vis Spectrophotometer

**Figure 1:**
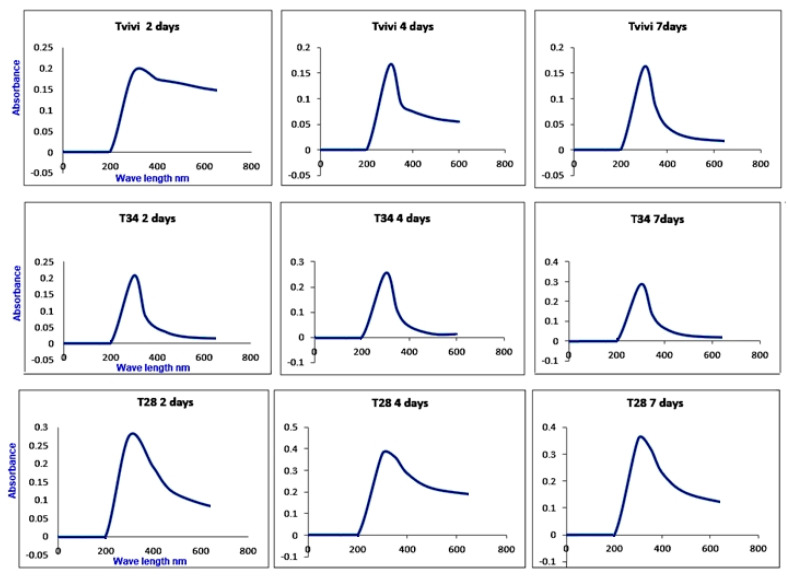
UV_vis spectrum of Trichogenic ZnONPs produced by Tvivi, T34 and T28 after 2, 4 and 7 days after synthesis.

Figure 1 shows the UV-vis absorption spectrum of zinc oxide nanoparticles. The absorption spectrum was recorded for the synthesized ZnONPs sample by Tvivi, TC 34 and TC 28 after 2, 4 and 7 days after synthesis in the range of 0 to 1100 nm. The absorbance peak at 300 nm, which corresponded to the distinctive band of ZnONPs, was visible in the spectrum, for all tested *Trichoderma* isolates at all tested days (Figure 1). ZnONPs produced by Tvivi strain was chosen for further characterization.

#### 3.3.2. Zeta Potential Analysis

**Figure 2:**
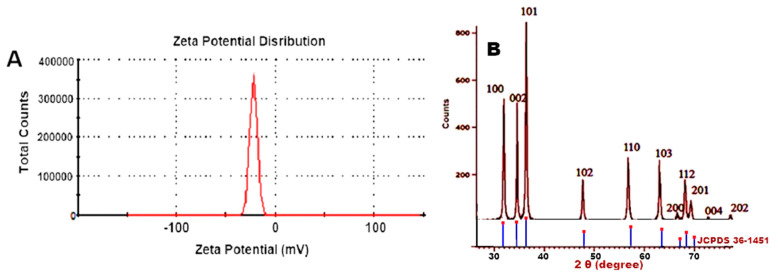
(**A**) Zeta potential analysis of synthesized ZnONPs. Trichoderma-mediated ZnONPs were spherical and rod-shaped and the potential value was found to be –24.0 mV. (**B**) X-ray diffraction pattern of Trichogenic ZnONPs. All peaks reveal the purity and crystalline nature. No traces of other impurity phases were detected.

The surface charges gained by ZnONPs were detected using zeta potential analysis, which may be used to learn more about the stability of the colloidal ZnONPs.In the present assay, we used a concentration of 40 μg/mL to measure zeta potential. As a result of this propensity, certain nanoparticles tended to agglomerate, reducing their surface area. ZnONPs must undergo prolonged ultrasonication in a water bath for at least 15 min to fix this problem. The result also signifies the presence of repulsive electrostatic forces among the synthesized ZnONPs, which leads to the monodispersity of the particles. In the present study, the zeta potential of ZnONPs was measured and was recorded as −24.0 mV (Figure 2A).

#### 3.3.3. X-ray Powder Diffractometer (XRD)

The XRD pattern of synthesized ZnONPs gave the diffraction peaks at (100), (002), (101), (102), (110), (103), (200), (112), (201), (004) and (202) planes, respectively, with the highest peak being the (101) plane (Figure 2B). The observed XRD peaks in the X-ray diffraction patterns of the ZnO samples were categorized by the hexagonal wurtzite structure of ZnO (JCPDS card 36-1451 data).

#### 3.3.4. Transmission Electron Microscopy (TEM)

**Figure 3:**
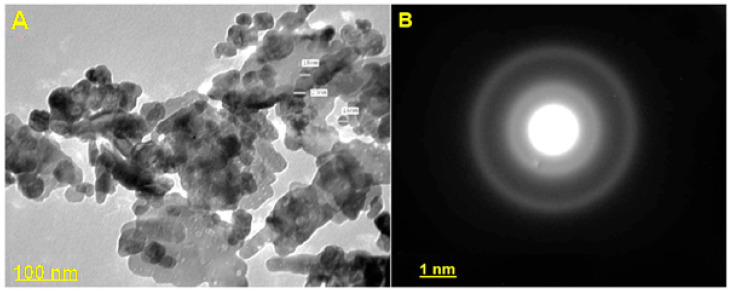
(**A**) Transmission electron microscopy (TEM) image of synthesized ZnO-NPs; the inset shows the corresponding particle size distribution and shape. (**B**): Selected Area Electron Diffraction (SAED) of Trichogenic-ZnONPs.

TEM was applied to know the actual size and shape of ZnONPs. The TEM image in the present study showed a mixture of hexagonal, spherical and rod-shaped a very small particles with a crystalline structure for the ZnONPs with an average size of 8–25 nm (Figure 3A). The ZnONPs were individuals and agglomerated in clusters. Diffraction rings could be allocated as (100), (002) and (101) planes from the selected area diffraction (SAED) pattern of ZnONPs (Figure 3B), representing hexagonal structure coupled with the wurtzite-like structure of ZnONPs as shown in the XRD pattern.

#### 3.3.5. Scanning Electron Microscopy (SEM)

**Figure 4:**
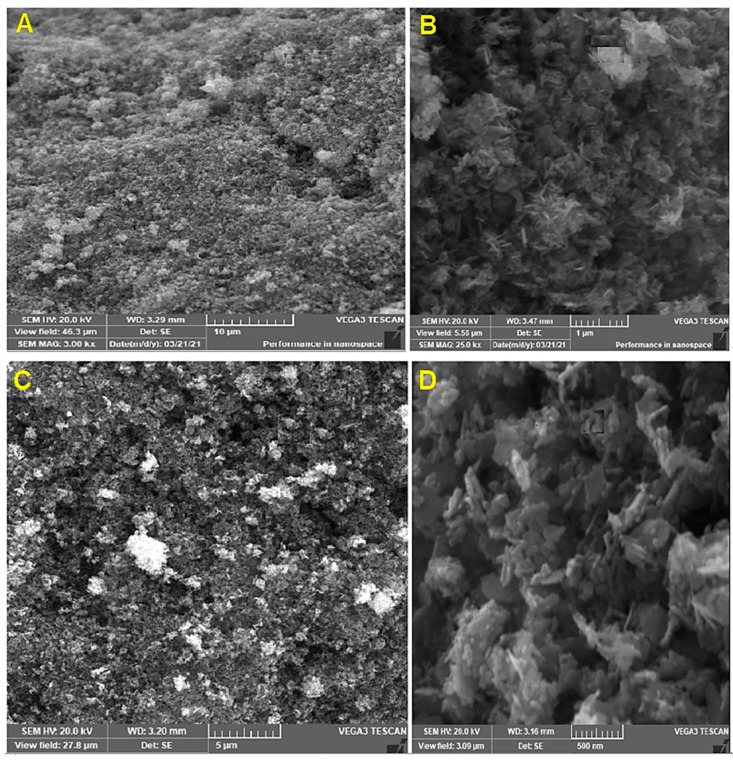
Scanning electron microscope micrographs at different magnifications, (**A**) 10, (**B**) 5, (**C**) 1µm and (**D**) 500 nm of Trichogenic-ZnONPs.

SEM is a high-resolution surface imaging approach that uses an electron beam to obtain information on nanostructures and other materials at the microscopic level. SEM analysis of synthesized ZnONPs exhibited clear spherical, rod and hexagonal shapes with and well-distributed ZnONPs with aggregation. Figure 4 presents a microscopic image of the obtained ZnONPs shown at different magnifications. The studies of the nanomaterial showed different sizes of the particles in a range comprising 24 to 50 nm. The microstructure of nanocrystalline ZnO had a skeletal form resulting from the process of coagulation (Figure 4A,B). The ZnONPs (Figure 4C,D).

#### 3.3.6. Energy Dispersive X-ray Analysis (EDX)

**Figure 5:**
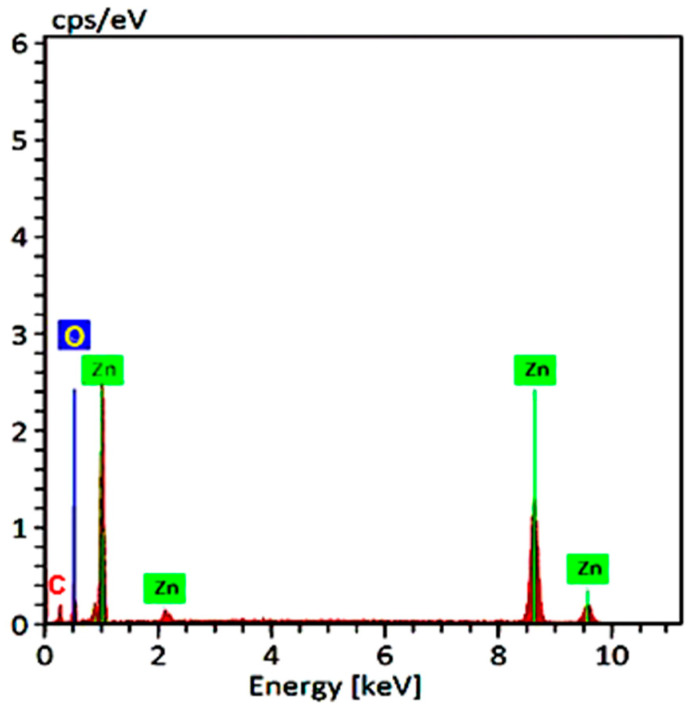
Elemental and energy dispersive X-ray spectroscopic analysis of Trichogenic-ZnONPs.

An EDX spectrum was used on the ZnONPs to determine the amount of metal and oxides in the sample. The EDX spectrum of the produced NPs was recorded in the spot-profile mode from one of the densely populated ZnONPs areas, as shown in Figure 5. The synthesis of ZnONPs is represented by distinct peaks observed for zinc and oxygen and carbon atoms. The atomic percentages of the elements inset of Figure 5 indicated zinc as the dominant element, representing more than 72.49% of the entire composition, with oxygen representing 27.51%, indicating that the ZnONPs were extremely pure.

#### 3.3.7. Fourier Transforms Infrared Spectroscopy (FTIR) Analysis

The interfaces between zinc oxide and bioactive components of fungal extract were discovered using FTIR on green synthesized ZnONPs. It was carried out to discover the organic functional groups or potential biomolecules involved in the production of ZnONPs. In the present results, FTIR spectrum showed 3398, 3233, 2912, 1640, 1629, 1561, 1461, 1018, 576 and 533 cm^−1^ (Table 2). In FTIR spectrum, the peak observed at 3398 cm^−1^ corresponded to OH stretching vibrations and 3233, peak observed at 3323 responding to C-H stretch of alkenyl and 1640 corresponded to C=O stretching 1629 responding to –C=C– aromatic stretching of fungal biomass and 1561 responding to C=C stretch in the aromatic ring and C=O stretch in polyphenols and 1461 corresponded to C-N stretch of amide-I in protein and 1018 responding to C-O stretching in amino acid, while 576 and 533 corresponded Zn-O stretching and hexagonal phase ZnO respectively.

### 3.4. In Vitro Antifungal Activity of Synthesized ZnONPs

**Figure 6:**
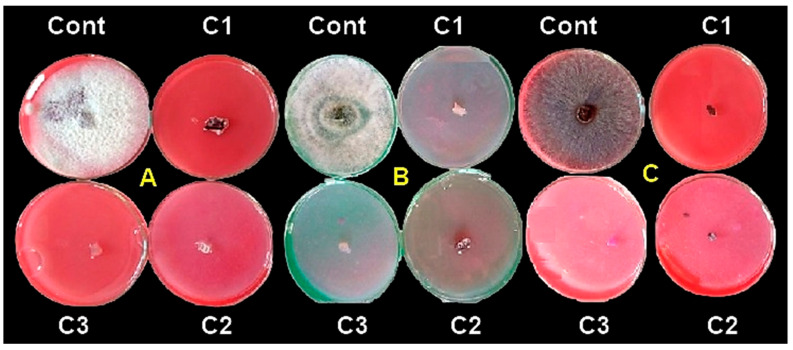
The inhibitory effect of mycelia growth on F10 (**A**), Rs9 (**B**), M4 (**C**)on potato dextrose agar medium containing ZnONPs at concentrations: Control, (**C1**) 20, (**C2**) 40 and (**C3**) 100 μg/mL after 7 days.

The potentiality of ZnONPs for controlling *R. solani* (RS9), *Fusarium* sp. (F10) and *M. phaseolina* (M4) was tested by plating the fungal culture media supplemented with (control), 20, 40 and 100 μg/mL of ZnONPs and the diameter of the mycelium growth was measured after 7 days. ZnONPs caused a significant reduction in the mycelia growth of *R. solani* (RS9), *Fusarium* sp. (F10) and *M. phaseolina* (M4) by all concentrations. As shown in (Figure 6), under the effect of ZnONPs treatments, the mycelial diameter of *R. solani* (RS9), *Fusarium* sp. (F10) and *M. phaseolina* (M4) was reduced by 100% at all the tested concentrations.

### 3.5. Effect of ZnONPs against Cotton Damping-Off Disease under Greenhouse Conditions

The effects of ZnONPs on the cotton seedling disease were studied in a greenhouse experiment. In a greenhouse, four treatments were tested for their ability to reduce cotton seedling disease produced by three pathogenic isolates (F10, Rs9 and M4). The number of surviving seedlings increased. Analysis of variance (ANOVA) (Table 3) showed that the treatment was a very highly significant source of variation (*p* = 0.00) of all the tested variables. Fungus × treatment interaction was a very highly significant source of variation (*p* = 0.00) only in the case of plant height. The effect of fungus was a non-significant source of variation in all the tested variables.

Since there was no fungus × treatment interaction on survival (Table 4), the general mean was used to compare treatment means. Seeds of Giza90 treated with ZnONPs (200 μg/mL) showed the maximum efficiency in controlling disease regardless of fungus (91.111% survival). The difference between the general means of the tested fungi was non-significant.

Because cultivar × treatment interaction was significant on plant height (Table 5), an interaction LSD was calculated to compare treatment means within each tested fungus. All treatments were effective in controlling disease compared to the infested control. The high concentrations of ZnONPs showed the maximum efficiency in controlling the disease for all tested fungi (25.193, 26.433 and 24.767 cm) for F10, Rs9 and M4, respectively. Since there was no fungus × treatment interaction on dry weight (Table 6), the general mean was used to compare treatment means. Seeds of Giza90 treated with ZnONPs (200 μg/mL) showed the maximum efficiency in controlling disease regardless of fungus (2.200 g). The difference between the general means of the tested fungi was non-significant.

Analysis of variance (ANOVA) of Table 7 showed that treatment was a very highly significant source of variation (*p* = 0.00) of all the tested variables. Fungus and fungus × treatment interaction was non-significant sources of variation of all the tested variables.

**Figure 7:**
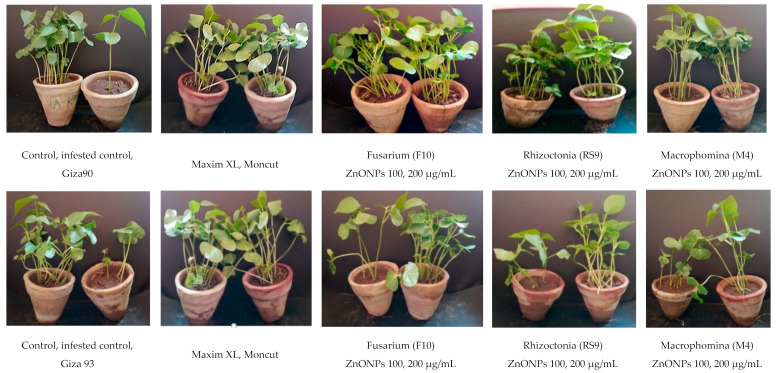
Cotton Seedlings cultivars Giza90 and Giza 93 obtained by sowing uncoated cotton seeds in sterilized soil infested with three fungal pathogens including, *Fusarium*, *R. solani* and *M. phaseolina* as a negative control, uncoated seeds sown in sterilized soil as a positive control, treated seeds with two fungicides (Maxim XL, Moncut) sown in infested soil and coated seeds with ZnONPs (100, 200 μg/mL) in infested soil. Photos were taken after 45 days under standard growth conditions in greenhouse conditions.

Since there were no effects of fungus × treatment interaction on survival (Table 8), the general mean was used to compare treatment means. Seeds of Giza94 treated with Moncut (2 g) showed the maximum efficiency in controlling disease regardless of fungus (88.889% survival) followed by Maxim XL (2 mL) and ZnONPs (200 μg/mL). The difference between the general means of the tested fungi was non-significant (Figure 7).

Since there was no fungus × treatment interaction on plant height and dry weight (Table 9 and Table 10), the general mean was used to compare treatment means. Seeds of Giza94 treated with ZnONPs (200 μg/mL) showed the maximum efficiency in controlling disease regardless of fungus (24.300 cm and 2.094 g). The difference between the general means of the tested fungi was non-significant.

## 4. Discussion

Nanoparticles derived from Trichoderma are still in the early stages of research. Mycogenic ZnONPs utilizing *Trichoderma* sp. are more compelling and less harmful to the environment than other methods. Therefore, ZnONPs produced utilizing a cell-free aqueous filtrate of *T. harzianum* were shown to have strong antifungal efficacy against the soil-borne pathogen complexes in cotton in this investigation. To confirm whether the synthesized nanoparticles were still stable for one week or changed in the UV results, the absorption spectrum was recorded for the synthesized ZnO sample by Tvivi, T34 and T28 after 2, 4 and 7 days after synthesis. The UV-visible spectrum showed the absorbance peak at 300 nm corresponding to the characteristic band of zinc oxide nanoparticles for all screened Trichoderma isolates at all tested periods. The obtained UV-vis spectrophotometer results were in agreement with Dobrucka et al. [33], who reported that the maximum absorption of about 310 nm, which is a characteristic band of pure ZnO, verified the presence of ZnONPs biologically with the use of the extract of *Chelidonium majus*. Furthermore, there was no additional peak in the spectrum, confirming that the produced products were pure ZnO [34,35]. In addition, Perveen et al. [36] reported that UV-visible spectroscopy investigation showed a peak at 300 nm, which corresponded to the wavelength of ZnO quantum dots’ surface plasmon resonance. The UV-vis spectra of ZnONPs synthesized by *A. niger* show that at 390 nm, ZnONPs have a high absorption spectra [37]. Jamdagni et al. [38] found that the UV spectrum range of ZnONPs is 320–390 nm, which is a similar result.

The XRD diffraction peaks were 31.84°, 34.52°, 36.33°, 47.63°, 56.71°, 62.96°, 68.13°, 69.18°, 70.16°, 73.21° and 78.56°, which agreed with Sadatzadeh et al., Yedurkar et al. and Malaikozhundanet al. [39,40,41]. The peaks showed the characteristic hexagonal wurtzite structure of ZnO (JCPDS card no. 36–1451) [42]. The Wurtzite structure was prevalent because it is stable in ambient conditions. It also revealed that the synthesized nanopowder was impurity-free because it lacked any XRD peaks other than zinc oxide peaks. The XRD diffraction peaks matched well with Wurtzite ZnO of the Joint Committee on Powder Diffraction Standards (JCPDS) Card number 36–1451 and were in good accord with the reported literature [43].

The magnitude of the Zeta potential (−30 mV to +30 mV) indicates the potential stability of the colloidal system [44,45,46]. The Zeta potential is related to the nanoparticles’ stability in the solution. The larger zeta potential values represent a lower degree of aggregation that leads to a higher degree of stability of nanoparticles and a smaller z-averaged hydrodynamic diameter. At lower zeta values, the nanoparticles flocculate early and the stability of the nano-suspension reduces [44]. The Zeta potential of ZnONPsin the present study was –24.0 mV, which provided evidence that the fabricated nanoparticles were moderately stable, which led to the monodispersity of the particles. The result was in agreement with Divya et al. [45], who showed a zeta potential of −5.36 mV. Furthermore, Zakharova [46] reported a zeta potential of 9 mV of ZnONPs had high antimicrobial efficacy and increased ZnONPs toxicity. It has been proven that some nanoparticles have a tendency to aggregate and that this process of aggregation reduces the surface area of nanoparticles. To solve this problem, ZnONPs require extensive ultrasonication in the water bath for a minimum of 15 min.

The results were in agreement with González et al. [47], who reported that TEM analysis of the synthesized ZnONPs showed spherical, hexagonal and rod shapes. Pillai et al. [48] reported that the synthesized ZnONPs from an aqueous extract of *Beta vulgaris* were spherical with a size of nearly 20 ± 2 nm. Morphology of bio nanoparticles produced from *Cinnamomum tamala* was rod-shaped, the particles size within the range 30 ± 3 nm.

TEM results of biosynthesized ZnONPs by *Anacardium occidentale* leaf extract confirmed the hexagonal structure with an average particle size of 33 nm [49]. Our results are in harmony with Ruddaraju et al. [50]. The results were in agreement with Ruddaraju et al. and Javed et al. [50,51]. The SEM images described surface topological details of different nano-objects based on the electron density of the surface due to their higher resolution and bigger field depth [52]. The agglomeration of ZnONPs might be attributed either due to its polarity and electrostatic attraction between ZnONPs or due to the high surface energy of ZnONPs. The high surface energy of ZnONPs could be originated from an aqueous synthetic medium [53,54,55]. TEM is used to magnify an image by using electromagnetic lenses to magnify an electron beam that travels through thin specimens in a nearly parallel manner. The objective lens is the principal electromagnetic lens. For example, an SEM picture generally shows bigger agglomerated particles, but TEM images have a greater resolution. This means that TEM is superior to SEM in terms of its ability to measure the nanoparticles’ size and has a greater resolution than SEM [54]. EDX analysis is a chemical microanalysis technique that is used in conjunction with SEM to evaluate elemental composition by detecting X-rays released from the sample during electron beam bombardment [55]. EDX analysis was in good agreement with XRD results. The EDX results of the present study were in agreement with several reports [36,39,40]. The FTIR spectrum revealed 3398, 3233, 2912, 1640, 1629, 1561, 1461, 1018, 576 and 533 cm^−1^ in the current study. The peak at 1640 corresponded to C=O stretching of the functional group. The peak in the range 1556 corresponded to C=C/amine—NH stretching of the aromatic compound [55].

**Figure 8:**
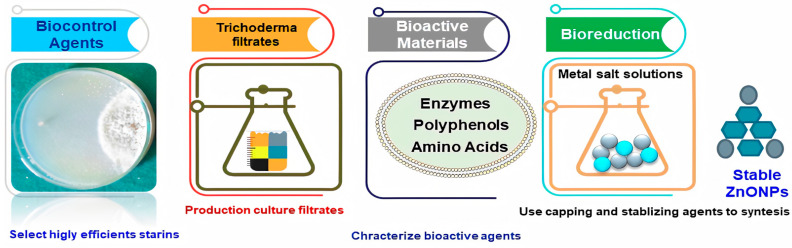
A schematic illustration depicting the methods used by *Trichoderma harzianum* strains to produce green zinc oxide nanoparticles.

The wide peak at 3233 cm^−1^ may be attributed to an alkenyl group’s C-H stretch, whereas 2104 cm^−1^ was moved to –C≡C– stretching vibrations [40]. Secondary metabolites found in *C. roseus* have been linked to the conversion of zinc acetate dihydrate to zinc oxide nanoparticles. The FTIR spectrum showed peaks at 3233, 2104, 1640, 1556, 1399, 1086, 926, 773, 849, 715, 1035, 482, 410 cm^−1^ [56]. Due to stretching alkenyl groups formed by zinc acetate salts and their reduction in ZnONPs, the FTIR spectra peak showed high-intensity broadband of 3233 cm^−1^ [37,56]. According to the results of our FTIR analysis, *Trichoderma*-mediated ZnONPs were synthesized using two distinct processes: reduction and capping. On the surfaces of both the biosynthesized ZnONPs that function as reducing and stabilizing agents, FTIR examination indicated the presence of proteins, amino acids, polyphenols, carboxyl and hydroxyl groups. ZnONPs are characterized by their strong aromatic ring and carboxylic acid appearance in the FTIR bands. According to the results of the FTIR analysis described various mycochemicals such as phenolic, proteins, amino acids, aldehydes, ketone and other functional groups were involved in the reduction, capping and stabilization of zinc oxide NPs (Figure 8).

In in vitro assay, in addition to inhibiting the vegetative mycelial growth of phytopathogenic fungi, zinc-mediated nanoparticles or composites can kill spores or inhibit spore germination (sporostatic/sporicidal activities) at low concentrations, such as a significant decrease in fungal growth of *B. cinerea* and *P. expansum* shown on ZnONPs (3 mM/L concentration) treatment [57]. Yehia and Ahmed [58] reported the antifungal efficiency of ZnONPs investigated against *F. oxysporum.* The maximum inhibition of mycelial growth was seen at (12 mg/L) when *F. oxysporum* growth was inhibited by 77 percent. HPLC quantification was used to study the influence of ZnONPs on the mycotoxin fusaric acid. The amount of fusaric acid was lowered from 39.0 to 0.20 mg/g. Scanning electron microscopy showed evident deformation in mycelia that had been treated with ZnONPs in *F. oxysporum*, which may cause growth inhibition.

In the present work, in vitro assay, zero fungal growth was investigated with concentrations starting from 20 μg/mL of ZnONPs. Fungicidal properties against three pathogenic fungi were explored in our study. Due to the current dearth of understanding of different aspects of fungal disease biology, these antifungal properties are currently restricted. Lahuf et al. [24] found that a concentration of 15 mg/mL led to complete inhibition (100%) of *R. solani*; however, lower doses of ZnONPs (10, 5 and 2.5 mg/mL) resulted in lower levels of inhibition of *R. solani*, by 83.21, 71.03 and 57 percent inhibition, respectively. Furthermore, it was discovered that ZnONPs have fungistatic rather than deadly fungicidal effects on *R. solani.* The ZnONPs fungicidal properties revealed that they were diffusible via the growing media [59]. Shen et al. and Raghupathi et al. [60,61] documented the antifungal effects of ZnONPs on microbial populations. It was suggested that zero-valent metal nanoparticles might successfully permeate pathogenic microorganism cell membranes through the lipid bilayer because of their reduced hydrophobicity due to the absence of surface charge [62]. ZnO showed obvious destruction of the cell walls and plasmolysis of the internal organs of the tested fungi [63]. In vitro studies against *F. oxysporum*, *R. solani* and *Sclerotium rolfsii* revealed that a mixture of *Trichoderma asperellum* and chitosan nanoparticles was better than *Trichoderma* alone and carbendazim 0.1% in suppressing pathogen mycelial growth [64].

In the current study, under greenhouse conditions, the results of the disease management studies of zinc oxide NPs, at two different concentrations (100 and 200 μg/mL), seed treatments for efficacy in the control of damping-off in cotton, compared to Maxim XL and Moncut chemical fungicides, indicated that ZnONPs (200 μg/mL), gave the maximum efficiency in disease control, compared to other treatments in Giza90 for all growth parameters (survival, plant height and dry weight). However, in the case of Giza94 cultivars, ZnONPs (200 μg/mL) NPs were not the best treatment in disease control in the case of survival only. However, it increased the survival significantly compared to infested control, but it was the best treatment in the case of plant height and dry weight. These results indicated that ZnONPs behavior was affected by the cultivar and it may need to be used at different optimum concentrations according to cotton cultivars to give the maximum survival during further future studies. The ZnONPs may form an antifungal layer around cotton seeds that protects cotton seedlings from the three pathogenic fungi. When ZnONPswas used as an antifungal agent against *R. solani* at concentrations of 30, 60 and 90 g ml^−1^, the second and the third concentration raised the percentages of Giza90 seedlings that survived to 85 and 86%, respectively, compared to 43.5 percent persisted seedlings at the concentration of 30 g m^−1^ [65]. González-Merino et al. [66] evaluated the antifungal activity of ZnONPs against *F.oxysporum* on tomato plants under greenhouse conditions. ZnONPs from 1500 to 3000 g/mL achieved the best plant height with a range of 166.0 to 175.40 cm, a severity of 0.40–0.80 and a disease incidence of 20–40%. In a pot experiment, foliar spraying of ZnONPs was more successful than seed priming in enhancing plant dry weight and controlling the *Pectobacterium betavasculorum*, *Meloidogyne incognita* and *R. solani*, causal disease complex of beetroot *(Beta Vulgaris* L.) [67]. Nevertheless, most ZnONPs may have accumulated on the seed’s exterior surface, with only a few particles moving into the stele and available for biodistribution and bioaccumulation, making seed priming less effective than foliar spray [68]. ZnONPs are believed to interact with pathogens through mechanical enfolding, which could be one of the main mechanisms of ZnONPs toxicity against *R. solani* [69].

## 5. Conclusions

This study used the biological control agent *T. harzianum* as a stabilizing agent for the green synthesis of biogenic ZnONPs with a relatively small size of 8–23 nm. UV-visible spectroscopy, XRD, zeta potential, TEM, SEM and EDX were used to validate the synthesis and structure, as well as to characterize size distribution, zeta potential, morphology and so on. Moreover, their antifungal activity against soil-borne pathogens like *R. solani* (RS9), *Fusarium* sp. (F10) and *M. phaseolina* (M4) were demonstrated in vitro and under greenhouse conditions. Trichogenic-mediated ZnONPs inhibited hyphal development in three cotton seedlings, indicating that they are effective against fungal infections. As a consequence of the aforementioned findings, it may be inferred that some *T. harzianum* strains produce a variety of proteins and enzymes, obviating the need for chemical reducers and stabilizers. As a result, the biological method for the production of ZnONPs utilizing *T. harzianum* has been presented in this work. The application of ZnONPs in the form of nanofungicides in agroecosystems has yet to be completely investigated and further study on risk assessment is still needed.

## Figures and Tables

**Table 1:** Fungicides and Trichogenic-ZnONPs applied in controlling damping-off of cotton seedlings under greenhouse conditions.

Treatment	Application Methods	Active Ingredients	Rate of Application
1-autoclaved soil	Seed dressing	Untreated	1 g for *R. solani* and 50 g for *Fusaruim* and *M. phaseolina* sterilized sorghum/kg soil
2-infested soil	Seed dressing	Untreated	1 g for *R. solani* and 50 g for *Fusaruim* and *M. phaseolina* infested sorghum/kg soil
3-Moncut	Seed dressing	Flutolanil	2 g/kg seeds
4-Maxim XL	Seed dressing	Fludioxonil, Mefanoxam	2 mL/L
5-ZnONPs	Seed dressing	Zinc Oxide	100 μg/mL
6-ZnONPs	Seed dressing	Zinc Oxide	200 μg/mL

**Table 2:** Functional Group present in the Trichogenic ZnONPs analyzed by FTIR.

	Frequency (cm) ^−1^	Abs	Possible Assignment
1	3398.9229	96.32	OH stretching vibrations
2	3233.0747	97.1775	C-H stretch of alkanyle
3	2912.9492	97.6787	The C-H stretch in alkanes
4	2854.1311	97.3885	O-H stretch in a carboxylic acid
5	1640.1611	99.027	C=O stretching
6	1629.5546	99.0942	–C=C– aromatic stretching of fungal biomass
7	1613.1626	98.9696	H-O-H binding vibration
8	1561.094	98.5039	C=C stretch in the aromatic ring and C=O stretch in polyphenols
9	1556.2728	98.4978	C=C/amine—NH stretching
10	1461.778	98.8046	C-N stretch of amide-I in protein
11	1382.7108	99.117	Acetate group stretching
12	1034.6226	96.4734	O-H Asymmetric stretching
13	1018.2305	96.3747	C-O stretching in amino acid
14	929.5211	97.4376	C-N stretching amine
15	773.3152	98.6183	C-N stretching amine
16	576.6116	95.2955	Zn-O stretching
17	533.2211	88.8474	hexagonal phase ZnO

**Table 3:** Analysis of variance of the effect of some fungi, treatments and their interaction on some growth variables of cotton seedlings of Giza90 grown in soil infested under greenhouse conditions.

Growth Variables and Sources of Variation	D.F	Mean Square	F Value	*p* ≥ F
Survival				
Replicates	2	118.51	0.51	0.61
Fungi (F)	2	78.65	0.34	0.72
Treatments (T)	5	3944.66	16.91	0.00
F × T	10	127.82	0.55	0.84
Error	34	23.22		
Plant height				
Replicates	2	3.72	0.31	0.74
Fungi (F)	2	17.95	1.49	0.24
Treatments (T)	5	244.83	20.25	0.00
F × T	10	50.89	4.21	0.00
Error	34	12.09		
Dry weight	2			
Replicates	2	0.22	0.90	0.42
Fungi (F)	5	0.32	1.30	0.29
Treatments (T)	10	4.13	16.7	0.00
F × T	34	0.23	0.94	0.51
Error	2	0.25		

**Table 4:** Effect of some fungi, treatments and their interaction on survival percentage of cotton seedlings of Giza90 grown in infested soil under greenhouse conditions.

ZnO/Survival/Giza90 (%)								
Treatment	F10		Rs9		M4		Mean	
	%	Transformed ^a^	%	Transformed ^a^	%	Transformed ^a^	%	Transformed ^a^
ZnONPs (μg/mL)	76.667	61.910	80.000	63.440	53.333	46.910	70.000	57.420
ZnONPs (μg/mL)	96.667	83.853	93.333	81.147	83.333	70.077	91.111	78.359
Maxim XL (2 mL)	86.667	72.293	86.667	72.783	90.000	75.000	87.778	73.359
Moncut (2 g)	86.667	72.783	83.333	70.763	83.333	66.147	84.444	69.898
Infested soil	30.000	28.077	16.667	15.000	26.667	30.293	24.445	24.457
Autoclaved soil	30.000	28.077	16.667	15.000	26.667	30.293	24.445	24.457
Mean	77.778	65.653	76.111	64.498	71.667	61.596	75.185	63.916

LSD (*p* ≤ 0.05) (transformed data) for treatments = 14.25. LSD (*p* ≤ 0.05) for fungus is non-significant. ^a^ Percentage of data were transformed into arcsine angles before carrying out the analysis of variance to produce an approximately constant variance.

**Table 5:** Effect of some fungi, treatments and their interaction on plant height of cotton seedlings of Giza90 grown in infested soil under greenhouse conditions.

ZnO/Plant Height (cm)/Giza90				
Treatment	F10	Rs9	M4	Mean
ZnONPs (100 μg/mL)	17.267	21.797	18.387	19.150
ZnONPs (200 μg/mL)	25.193	26.433	24.767	25.464
Maxim XL (2 mL)	18.573	21.843	15.917	18.778
Moncut (2 g)	20.147	16.627	21.137	19.304
Infested soil	7.150	4.800	20.303	10.751
Autoclaved soil	24.307	24.833	23.847	24.329
Mean	18.773	19.389	20.726	19.629

LSD (*p* ≤ 0.05) for fungus ×treatment = 5.62.

**Table 6:** Effect of some fungi, treatments and their interaction on the dry weight of cotton seedlings of Giza90 grown in soil infested under greenhouse conditions.

ZnO/Dry Weight (g)/Giza90				
Treatment	F10	Rs9	M4	Mean
ZnONPs (100 μg/mL)	1.700	1.493	1.370	1.521
ZnONPs (200 μg/mL)	2.250	2.377	1.973	2.200
Maxim XL (2 mL)	2.073	1.527	1.137	1.579
Moncut (2 g)	1.660	1.833	1.137	1.543
Infested soil	0.187	0.133	0.377	0.232
Autoclaved soil	1.660	2.050	2.090	1.933
Mean	1.588	1.569	1.347	1.501

LSD (*p* ≤ 0.05) for treatments = 0.47. LSD (*p* ≤ 0.05) for fungus is non-significant.

**Table 7:** Analysis of variance of the effect of some fungi, treatments and their interaction on some growth variables of cotton seedlings of Giza94 grown in soil infested under greenhouse conditions.

Growth Variables and Sources of Variation	D.F.	Mean Square	F. Value	*p* ≥ F
Survival				
Replicates	2	556.02	2.79	0.08
Fungi (F)	2	19.16	0.10	0.91
Treatments (T)	5	4674.53	23.45	0.00
F × T	10	215.36	1.08	0.40
Error	34	199.32		
Plant height				
Replicates	2	38.05	1.37	0.27
Fungi (F)	2	23.36	0.84	0.44
Treatments (T)	5	320.18	11.52	0.00
F × T	10	36.91	1.33	0.26
Error	34	27.80		
Dry weight	2			
Replicates	2	0.42	1.32	0.28
Fungi (F)	5	0.14	0.46	0.64
Treatments (T)	10	4.99	15.77	0.00
F × T	34	0.33	1.04	0.43
Error	2	0.32		

**Table 8:** Effect of some fungi, treatments and their interaction on survival percentage of cotton seedlings of Giza94 grown in soil infested under greenhouse conditions.

ZnO/Survival/Giza94 (%)								
Treatment	F10		Rs9		M4		Mean	
	%	Transformed ^a^	%	Transformed ^a^	%	Transformed ^a^	%	Transformed ^a^
ZnONPs (100 μg/mL)	36.667	36.930	10.000	11.070	43.333	41.057	30.000	29.686
ZnONPs (200 μg/mL)	73.333	59.693	73.333	59.693	76.667	61.223	74.444	60.203
Maxim XL (2 mL)	80.000	67.860	76.667	65.840	76.667	61.910	77.778	65.203
Moncut (2 g)	83.333	70.077	93.333	77.707	90.000	75.000	88.889	74.261
Infested soil	16.667	19.223	30.000	28.077	6.667	12.293	17.778	19.864
Autoclaved soil	86.667	72.783	86.667	72.293	83.333	66.147	85.556	70.408
Mean	62.778	54.428	61.667	52.447	62.778	52.938	62.408	53.271

LSD (*p* ≤ 0.05) (transformed data) for treatments = 13.18. LSD (*p* ≤ 0.05) for fungus is non-significant. ^a^ Percentage data were transformed into arcsine angles before carrying out the analysis of variance to produce an approximately constant variance.

**Table 9:** Effect of some fungi, treatments and their interaction on plant height of cotton seedlings of Giza94 grown in infested soil under greenhouse conditions.

ZnO/Plant Height (cm)/Giza94				
Treatment	F10	Rs9	M4	Mean
ZnONPs (100 μg/mL)	19.527	4.887	14.830	13.081
ZnONPs (200 μg/mL)	25.377	22.557	24.967	24.300
Maxim XL (2 mL)	19.113	22.713	18.997	20.274
Moncut (2 g)	21.333	22.653	17.973	20.653
Infested soil	11.917	11.533	11.000	11.483
Autoclaved soil	26.730	26.950	25.507	26.396
Mean	20.666	18.549	18.879	19.365

LSD (*p* ≤ 0.05) for treatments = 4.92., LSD (*p* ≤ 0.05) for fungus is non-significant.

**Table 10:** Effect of some fungi, treatments and their interaction on the dry weight of cotton seedlings of Giza94 grown in infested soil under greenhouse conditions.

ZnO/Dry Weight (g)/Giza94				
Treatment	F10	Rs9	M4	Mean
ZnONPs (100 μg/mL)	1.427	0.507	1.563	1.166
ZnONPs (200 μg/mL)	2.253	2.170	1.860	2.094
Maxim XL(2 mL)	1.537	1.753	1.490	1.593
Moncut (2 g)	1.697	1.497	1.360	1.518
Infested soil	0.403	0.707	0.537	0.549
Autoclaved soil	2.837	3.020	2.270	2.709
Mean	1.692	1.609	1.513	1.605

LSD (*p* ≤ 0.05) for treatments = 0.53. LSD (*p* ≤ 0.05) for fungus is non-significant.

## References

[1] Kew Royal Botanic Gardens (1897). Cultivation of Cotton in Egypt. (*Gossypium barbadense*, L.). Bull. Misc. Inf. Kew.

[2] Asran-Amal A., Abd-Elsalam K.A., Omar M.R., Aly A.A. (2005). Antagonistic potential of *Trichoderma* spp. against *Rhizoctonia solani* and use of M13 microsatellite-primed PCR to evaluate the antagonist genetic variation. J. Plant Dis. Prot..

[3] Fulton N.D., Awaddle B., Thomas J.A. (1956). Influence of Planting Date on Fungi Isolated from Diseased Cotton Seedlings. Plant Dis. Rep..

[4] Colyer P.D. (1988). Frequency and Pathogenicity of *Fusarium* spp. Associated with Seedling Diseases of Cotton in Louisiana. Plant Dis..

[5] Mihail J.D., Taylor S.J. (1995). Interpreting Variability among Isolates of *Macrophominaphaseolina* in Pathogenicity, Pycnidium Production and Chlorate Utilization. Can. J. Bot..

[6] Srivastava A.K., Singh T., Jana T.K., Arora D.K. (2001). Induced Resistance and Control of Charcoal Rot in *Cicer Arietinum* (Chickpea) by *Pseudomonas fluorescens*. Can. J. Bot..

[7] Mihail J.D., Singleton L.L., Mihail J.D., Rush C.M. (1992). Macrophomina. Methods for Research on Soil-Borne Phytopathogenic Fungi.

[8] Khan S.A., Shahid S., Mahmood T., Lee C.-S. (2021). Contact Lenses Coated with Hybrid Multifunctional Ternary Nanocoatings (Phytomolecule-Coated ZnO nanoparticles: Gallic Acid: Tobramycin) for the Treatment of Bacterial and Fungal Keratitis. Acta Biomater..

[9] Abd-Elsalam K.A., Hashim A.F., Alghuthaymi M.A., Said-Galiev E. (2017). Nanobiotechnological Strategies for Toxigenic Fungi and Mycotoxin Control. Food Preservation.

[10] Shobha B., Lakshmeesha T.R., Ansari M.A., Almatroudi A., Alzohairy M.A., Basavaraju S., Alurappa R., Niranjana S.R., Chowdappa S. (2020). Mycosynthesis of ZnO Nanoparticles Using *Trichoderma* spp. Isolated from Rhizosphere Soils and Its Synergistic Antibacterial Effect against *Xanthomonas oryzae* pv. *oryzae*. J. Fungi.

[11] Yusof H.M., Mohamad R., Zaidan U.H., Rahman N.A.A. (2019). Microbial synthesis of zinc oxide nanoparticles and their potential application as an antimicrobial agent and a feed supplement in animal industry: A review. J. Anim. Sci. Biotechnol..

[12] Ammar H.A., Alghazaly M.S., Assem Y., AbouZeid A.A. (2021). Bioengineering and Optimization of PEGylated Zinc Nanoparticles by Simple Green Method Using *Monascus purpureus* and Their Powerful antifungal Activity Against the Most Famous Plant Pathogenic Fungi, Causing Food Spoilage. Environ. Nanotechnol. Monit. Manag..

[13] Abd-Elsalam K.A., Khokhlov A.R. (2015). Eugenol Oil Nanoemulsion: Antifungal Activity against *Fusarium oxysporum* f. sp. *vasinfectum* and Phytotoxicity on Cottonseeds. Appl. Nanosci..

[14] Alghuthaymi M.A., Almoammar H., Rai M., Said-Galiev E., Abd-Elsalam K.A. (2015). Myconanoparticles: Synthesis and Their Role in Phytopathogens Management. Biotechnol. Biotechnol. Equip..

[15] Sosna-Głębska A., Sibiński M., Szczecińska N., Apostoluk A. (2020). UV–Visible Silicon Detectors with Zinc Oxide Nanoparticles Acting as Wave length Shifters. Mater. Today.

[16] Fahimmunisha B.A., Ishwarya R., AlSalhi M.S., Devanesan S., Govindarajan M., Vaseeharan B. (2020). Green Fabrication, Characterization and Antibacterial Potential of Zinc Oxide Nanoparticles Using *Aloe Socotrina* Leaf Extract: A Novel Drug Delivery Approach. J. Drug Deliv. Sci. Technol..

[17] Lingaraju K., Naika H.R., Manjunath K., Basavaraj R.B., Nagabhushana H., Nagaraju G., Suresh D. (2016). Biogenic Synthesis of Zinc Oxide Nanoparticles Using *Rutagraveolens* (L.) and Their Antibacterial and Antioxidant Activities. Appl. Nanosci..

[18] Hwa K.-Y., Subramani B. (2014). Synthesis of Zinc Oxide Nanoparticles on Graphene-Carbon Nanotube Hybrid for Glucose Biosensor Applications. Biosens. Bioelectron..

[19] Singh J., Dutta T., Kim K.H., Rawat M., Samddar P., Kumar P. (2018). Green’synthesis of Metals and Their Oxide Nanoparticles: Applications for Environmental Remediation. J. Nanobiotechnol..

[20] Pokhrel L.R., Dubey B. (2013). Evaluation of Developmental Responses of Two Crop Plants Exposed to Silver and Zinc Oxide Nanoparticles. Sci. Total Environ..

[21] Sur D.H., Mukhopadhyay M. (2019). Role of Zinc Oxide Nanoparticles for Effluent Treatment Using *Pseudomonas putida* and *Pseudomonas aureofaciens*. Bioprocess Biosyst. Eng..

[22] Sun Q., Li J., Le T. (2018). Zinc Oxide Nanoparticle as a Novel Class of Antifungal Agents: Current Advances and Future Perspectives. J. Agric. Food Chem..

[23] Kalia A., Abd-Elsalam K.A., Kuca K. (2020). Zinc-Based Nanomaterials for Diagnosis and Management of Plant Diseases: Ecological Safety and Future Prospects. J. Fungi.

[24] Lahuf A.A., Kareem A.A., Al-Sweedi T.M., Alfarttoosi H.A. (2019). Evaluation the Potential of Indigenous Biocontrol Agent Trichoderma Harzianum and Its Interactive Effect with Nanosized ZnO Particles against the Sunflower Damping-off Pathogen. *Rhizoctoniasolani*. Proceedings of the IOP Conference Series: Earth and Environmental Science.

[25] Khan S.A., Shahid S., Lee C.-S. (2020). Green Synthesis of Gold and Silver Nanoparticles Using Leaf Extract of *ClerodendrumInerme*; Characterization, Antimicrobial and Antioxidant Activities. Biomolecules.

[26] Consolo V.F., Torres-Nicolini A., Alvarez V.A. (2020). Mycosinthetized Ag, CuO and ZnO Nanoparticles from a Promising *Trichoderma harzianum* Strain and Their Antifungal Potential against Important Phytopathogens. Sci. Rep..

[27] Zhou C., Guo R., Ji S., Fan H., Wang J., Wang Y., Liu Z. (2020). Isolation of *Trichoderma* from Forestry Model Base and the Antifungal Properties of Isolate TpsT17 toward *Fusarium oxysporum*. Microbiol. Res..

[28] Dou K., Gao J., Zhang C., Yang H., Jiang X., Li J., Li Y., Wang W., Xian H., Li S. (2019). *Trichoderma* biodiversity in major ecological systems of China. J. Microbiol..

[29] Elamawi R.M., Al-Harbi R.E., Hendi A.A. (2018). Biosynthesis and Characterization of Silver Nanoparticles Using *Trichoderma Longibrachiatum* and Their Effect on Phytopathogenic Fungi. Egypt. J. Biol. Pest Control.

[30] Rajan A., Cherian E., Baskar G. (2016). Biosynthesis of zinc oxide nanoparticles using *Aspergillus fumigatus* JCF and its antibacterial activity. Int. J. Mod. Sci. Technol..

[31] Henam S.D., Ahmad F., Shah M.A., Parveen S., Wani A.H. (2019). Microwave Synthesis of Nanoparticles and Their Antifungal Activities. Spectrochim. Acta A Mol. Biomol. Spectrosc..

[32] Abd-Elsalam K.A., Vasil’kov A.Y., Said-Galiev E.E., Rubina M.S., Khokhlov A.R., Naumkin A.V., Shtykova E.V., Alghuthaymi M.A. (2018). Bimetallic Blends and Chitosan Nanocomposites: Novel Antifungal Agents against Cotton Seedling Damping-Off. Eur. J. Plant Pathol..

[33] Dobrucka R., Dlugaszewska J., Kaczmarek M. (2018). Cytotoxic and Antimicrobial Effects of Biosynthesized ZnO Nanoparticles Using of *Chelidonium majus* Extract. Biomed. Microdevices.

[34] Wahab R., Ansari S.G., Kim Y.S., Seo H.K., Kim G.S., Khang G., Shin H.-S. (2007). Low-Temperature Solution Synthesis and Characterization of ZnO Nano-Flowers. Mater. Res. Bull..

[35] Wahab R., Ansari S.G., Kim Y.S., Song M., Shin H.-S. (2009). The Role of PH Variation on the Growth of Zinc Oxide Nanostructures. Appl. Surf. Sci..

[36] Perveen R., Shujaat S., Qureshi Z., Nawaz S., Khan M.I., Iqbal M. (2020). Green versus Sol-Gel Synthesis of ZnO Nanoparticles and Antimicrobial Activity Evaluation against Panel of Pathogens. J. Mater. Res. Technol..

[37] Gao Y., Arokia Vijaya Anand M., Ramachandran V., Karthikkumar V., Shalini V., Vijayalakshmi S., Ernest D. (2019). Biofabrication of Zinc Oxide Nanoparticles from *Aspergillus niger*, Their Antioxidant, Antimicrobial and Anticancer Activity. J. Clust. Sci..

[38] Jamdagni P., Khatri P., Rana J.S. (2018). Green Synthesis of Zinc Oxide Nanoparticles Using Flower Extract of *Nyctanthes Arbor-Tristis* and Their Antifungal Activity. J. King Saud Univ. Sci..

[39] Sadatzadeh A., Charati F.R., Akbari R., Moghaddam H.H. (2018). Green Biosynthesis of Zinc Oxide Nanoparticles via Aqueous Extract of Cottonseed. J. Mater. Environ. Sci..

[40] Yedurkar S., Maurya C., Mahanwar P. (2016). Biosynthesis of Zinc Oxide Nanoparticles Using Ixora Coccinea Leaf Extract—A Green Approach. Open J. Synth. Theory Appl..

[41] Malaikozhundan B., Vinodhini J. (2018). Nanopesticidal Effects of *Pongamiapinnata* Leaf Extract Coated Zinc Oxide Nanoparticle against the Pulse Beetle, *Callosobruchus maculatus*. Mater. Today Commun..

[42] Soares V.A., Xavier M.J.S., Rodrigues E.S., de Oliveira C.A., Farias P.M.A., Stingl A., Ferreira N.S., Silva M.S. (2020). Green Synthesis of ZnO Nanoparticles Using Whey as an Effective Chelating Agent. Mater. Lett..

[43] Pandao M.R., Sajid M. (2021). Synthesis and Characterization of Nano Zinc Oxide for Linseed. J. Pharmacogn. Phytochem..

[44] Baddar Z.E., Matocha C.J., Unrine J.M. (2019). Surface coating effects on the sorption and dissolution of ZnO nanoparticles in soil. Environ. Sci. Nano.

[45] Divya M., Govindarajan M., Karthikeyan S., Preetham E., Alharbi N.S., Kadaikunnan S., Khaled J.M., Almanaa T.N., Vaseeharan B. (2020). Antibiofilm and Anticancer Potential of β-Glucan-Binding Protein-Encrusted Zinc Oxide Nanoparticles. Microb. Pathog..

[46] Zakharova O., Kolesnikov E., Vishnyakova E., Strekalova N., Gusev A. (2019). Antibacterial activity of ZnO nanoparticles: Dependence on particle size, dispersion media and storage time. Proceedings of the IOP Conference Series: Earth Environmental Science.

[47] González S.C.E., Bolaina-Lorenzo E., Pérez-Trujillo J.J., Puente-Urbina B.A., Rodríguez-Fernández O., Fonseca-García A., Betancourt-Galindo R. (2021). Antibacterial and Anticancer Activity of ZnO with Different Morphologies: A Comparative Study. 3 Biotech.

[48] Pillai A.M., Sivasankarapillai V.S., Rahdar A., Joseph J., Sadeghfar F., Anuf A.R., Rajesh K., Kyzas G.Z. (2020). Green Synthesis and Characterization of Zinc Oxide Nanoparticles with Antibacterial and Antifungal Activity. J. Mol. Struct..

[49] Zhao C., Zhang X., Zheng Y. (2018). Biosynthesis of Polyphenols Functionalized ZnO Nanoparticles: Characterization and Their Effect on Human Pancreatic Cancer Cell Line. J. Photochem. Photobiol. B.

[50] Ruddaraju L.K., Pammi S.V.N., Pallela P.N.V.K., Padavala V.S., Kolapalli V.R.M. (2019). Antibiotic Potentiation and Anti-Cancer Competence through Bio-Mediated ZnO Nanoparticles. Mater. Sci. Eng. C Mater. Biol. Appl..

[51] Javed R., Rais F., Fatima H., Haq I.U., Kaleem M., Naz S.S., Ao Q. (2020). Chitosan Encapsulated ZnO Nanocomposites: Fabrication, Characterization and Functionalization of Bio-Dental Approaches. Mater. Sci. Eng. C Mater. Biol. Appl..

[52] Elumalai K., Velmurugan S., Ravi S., Kathiravan V., Adaikala Raj G. (2015). Bio-Approach: Plant Mediated Synthesis of ZnO Nanoparticles and Their Catalytic Reduction of Methylene Blue and Antimicrobial Activity. Adv. Powder Technol..

[53] Teulon J.M., Godon C., Chantalat L., Moriscot C., Cambedouzou J., Odorico M., Ravaux J., Podor R., Gerdil A., Habert A. (2018). On the Operational Aspects of Measuring Nanoparticle Sizes. Nanomaterials.

[54] Nain V., Kaur M., Sandhu K.S., Thory R., Sinhmar A. (2020). Development, Characterization and Biocompatibility of Zinc Oxide Coupled Starch Nanocomposites from Different Botanical Sources. Int. J. Biol. Macromol..

[55] Rastogi L., Arunachalam J. (2011). Sunlight Based Irradiation Strategy for Rapid Green Synthesis of Highly Stable Silver Nanoparticles Using Aqueous Garlic (*Allium sativum*) Extract and Their Antibacterial Potential. Mater. Chem. Phys..

[56] Gupta M., Tomar R.S., Kaushik S., Mishra R.K., Sharma D. (2018). Effective Antimicrobial Activity of GreenZnONano Particles of *Catharanthus roseus*. Front. Microbiol..

[57] He L., Liu Y., Mustapha A., Lin M. (2011). Antifungal Activity of Zinc Oxide Nanoparticles against *Botrytis Cinerea* and *Penicillium expansum*. Microbiol. Res..

[58] Yehia R.S., Ahmed O.F. (2013). In Vitro Study of the Antifungal Efficacy of Zinc Oxide Nanoparticles against *Fusarium oxysporum* and *Peniciliumexpansum*. Afr. J. Microbiol. Res..

[59] Barsainya M., Pratap Singh D. (2018). Green Synthesis of Zinc Oxide Nanoparticles by *Pseudomonas aeruginosa* and Their Broad-Spectrum Antimicrobial Effects. J. Pure Appl. Microbiol..

[60] Shen Z., Chen Z., Hou Z., Li T., Lu X. (2015). Ecotoxicological Effect of Zinc Oxide Nanoparticles on Soil Microorganisms. Front. Environ. Sci. Eng..

[61] Raghupathi K.R., Koodali R.T., Manna A.C. (2011). Size-Dependent Bacterial Growth Inhibition and Mechanism of Antibacterial Activity of Zinc Oxide Nanoparticles. Langmuir.

[62] Hulkoti N.I., Taranath T.C. (2014). Biosynthesis of Nanoparticles Using Microbes—A Review. Colloids Surf. B Biointerfaces.

[63] Farouk A.M. (2016). Improvement of Bean Resistance to Damping-Off and Root-Rot Diseases. Ph.D. Thesis.

[64] Boruah S., Dutta P. (2021). Fungus Mediated Biogenic Synthesis and Characterization of Chitosan Nanoparticles and Its Combine Effect with *Trichoderma asperellum* against *Fusarium oxysporum, Sclerotium rolfsii* and *Rhizoctonia solani*. Indian Phytopathol..

[65] Al-Dhabaan F.A., Shoala T., Ali A.A., Alaa M., Abd-Elsalam K., Abd-Elsalam K. (2017). Chemically-Produced Copper, Zinc Nanoparticles and Chitosan-Bimetallic Nanocomposites and Their Antifungal Activity against Three Phytopathogenic Fungi. Int. J. Agric. Technol..

[66] González-Merino A.M., Hernández-Juárez A., Betancourt-Galindo R., Ochoa-Fuentes Y.M., Valdez-Aguilar L.A., Limón-Corona M.L. (2021). Antifungal Activity of Zinc Oxide Nanoparticles in *Fusarium oxysporum*—*Solanum lycopersicum* Pathosystem under Controlled Conditions. J. Phytopathol..

[67] Khan M.R., Siddiqui Z.A. (2021). Role of Zinc Oxide Nanoparticles in the Management of Disease Complex of Beetroot (*Beta vulgaris* L.) Caused by *Pectobacterium betavasculorum*, *Meloidogyne incognita* and *Rhizoctonia solani*. Hortic. Environ. Biotechnol..

[68] Khan M., Siddiqui Z.A. (2018). Zinc Oxide Nanoparticles for the Management of *Ralstoniasolanacearum*, *Phomopsis Vexans* and *Meloidogyne Incognita* Incited Disease Complex of Eggplant. Indian Phytopathol..

[69] Khan M., Khan A.U., Alam J., Parveen A., Moon I.-S., Alam M., Cabral-Pinto M.M., Ahamed M., Yadav V.K., Yadav K. (2021). Molecular Docking of Biosynthesized Zinc Oxide Nanoparticles to Screen Their Impact on Fungal Pathogen of Carrot Plant. Res. Sq..

